# Remimazolam‐Based Anesthesia Without Neuromuscular Blockade for Laparoscopic Surgery in a Patient With Immune‐Mediated Necrotizing Myopathy: A Case Report

**DOI:** 10.1155/cria/3589489

**Published:** 2026-09-21

**Authors:** Yuka Kobayashi, Ryo Wakabayashi, Kanji Uchida

**Affiliations:** ^1^ Department of Anesthesiology and Pain Relief Center, The University of Tokyo Hospital, 7-3-1 Hongo Bunkyo-ku, Tokyo 113-8655, Japan, u-tokyo.ac.jp

**Keywords:** case report, immune-mediated necrotizing myopathy, laparoscopy, neuromuscular blocking agents, remimazolam

## Abstract

**Background:**

Immune‐mediated necrotizing myopathy causes progressive muscle weakness and may complicate anesthetic management because responses to neuromuscular blocking agents may be unpredictable and residual blockade may further compromise respiratory function. We report a case in which laparoscopic surgery was completed without neuromuscular blocking agents under remimazolam‐based total intravenous anesthesia.

**Case Presentation:**

A 44‐year‐old woman with anti–3‐hydroxy‐3‐methylglutaryl‐coenzyme A reductase antibody–positive immune‐mediated necrotizing myopathy and marked preexisting muscle weakness underwent laparoscopic myomectomy under total intravenous anesthesia with remimazolam and remifentanil without neuromuscular blocking agents. Low‐dose rocuronium was immediately available as rescue treatment. Tracheal intubation was completed without coughing or movement. No patient movement occurred during pneumoperitoneum, and surgical exposure remained acceptable. Flumazenil was administered after responsiveness to verbal commands, airway reflexes, and adequate spontaneous ventilation had been confirmed. Tracheal extubation was uneventful, and no postoperative resedation or respiratory compromise occurred.

**Conclusion:**

In this patient with immune‐mediated necrotizing myopathy and marked preexisting muscle weakness, laparoscopic surgery was completed without neuromuscular blocking agents under remimazolam‐based total intravenous anesthesia, with low‐dose rocuronium available as rescue treatment. Tracheal intubation was smooth, surgical conditions were acceptable, and postoperative respiratory recovery was uncomplicated.

## 1. Introduction

Immune‐mediated necrotizing myopathy (IMNM) is an autoimmune myopathy characterized by progressive proximal muscle weakness; dysphagia and respiratory muscle involvement may also occur [1]. These manifestations are relevant to anesthetic management because reduced respiratory reserve may increase vulnerability to postoperative respiratory complications, particularly in the presence of residual neuromuscular blockade [2, 3]. In patients with neuromuscular disorders, responses to nondepolarizing neuromuscular blocking agents may be altered or difficult to predict [2, 3]. Although nondepolarizing neuromuscular blocking agents can be titrated using quantitative monitoring and reversed with sugammadex, evidence regarding sugammadex in patients with neuromuscular disease remains limited [4].

In selected patients, planned avoidance of neuromuscular blocking agents may be considered an individualized option. Laparoscopic surgery without neuromuscular blockade still requires adequate intubating conditions, patient immobility, and surgical exposure. Remimazolam is an ultrashort‐acting benzodiazepine hypnotic whose effects can be reversed with flumazenil [5]. We report remimazolam‐based total intravenous anesthesia without neuromuscular blocking agents for laparoscopic surgery in an adult with IMNM and marked preexisting muscle weakness. This report was prepared in accordance with the CARE guidelines.

## 2. Case Presentation

A 44‐year‐old woman (height, 157.6 cm; weight, 47.2 kg) was scheduled for laparoscopic myomectomy. She had developed muscle weakness at 32 years of age and was diagnosed with IMNM based on muscle biopsy findings. Her serum creatine kinase concentration was approximately 7000 U/L at diagnosis, and subsequent antibody testing identified her disease as anti–3‐hydroxy‐3‐methylglutaryl‐coenzyme A reductase antibody–positive IMNM. She was receiving prednisolone 3 mg/day and methotrexate 12 mg/week. Her condition had remained clinically stable during the year before surgery. Muscle strength was grade 2 on the Medical Research Council scale for neck flexion and extension and for the bilateral quadriceps and hamstrings. She also had gait unsteadiness and dysarthria.

The preoperative serum creatine kinase concentration was 452 U/L. Chest radiography and electrocardiography showed no abnormalities, and oxygen saturation measured by pulse oximetry was 98% while breathing room air. Pulmonary function testing and formal assessment of respiratory muscle strength were not performed. The anesthetic plan was to avoid neuromuscular blocking agents initially, with low‐dose rocuronium immediately available if intubation, patient immobility, or surgical exposure proved inadequate. If rocuronium was administered, quantitative neuromuscular monitoring and reversal with sugammadex were planned. This plan was agreed upon with the surgical team before induction.

Anesthesia was induced with remimazolam 12 mg/kg/h and remifentanil 0.25 μg/kg/min. A total of 23 mg of remimazolam was administered before tracheal intubation. After loss of consciousness, an electromyography‐based neuromuscular monitor (AF‐201P; Nihon Kohden, Tokyo, Japan) was calibrated at the right adductor pollicis. Laryngoscopy was performed without rocuronium. No coughing or patient movement occurred, and tracheal intubation was completed smoothly using a McGRATH MAC video laryngoscope (Medtronic, Minneapolis, MN) and a 7.0‐mm internal‐diameter tracheal tube.

**FIGURE 1:**
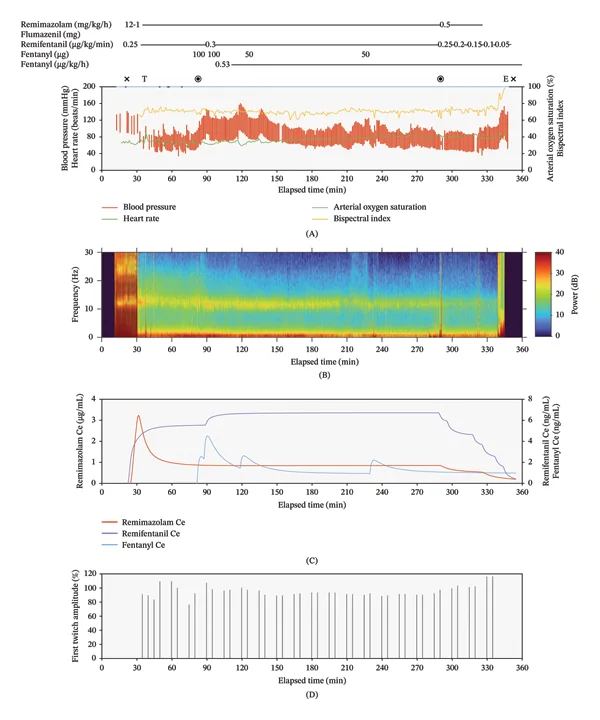
Perioperative course of remimazolam‐based anesthesia without neuromuscular blocking agents. (A) Time course of anesthetic and analgesic administration and intraoperative physiologic variables. ×, start and end of anesthesia; T, tracheal intubation; ◉, start and end of surgery; E, tracheal extubation. (B) Electroencephalographic spectrogram during anesthesia. Spectral power is displayed over the 0–30‐Hz frequency range. Activity was dominated by delta and alpha oscillations during maintenance of anesthesia. (C) Estimated effect‐site concentrations of remimazolam, remifentanil, and fentanyl, calculated using published pharmacokinetic/pharmacodynamic models [6–8]. Ce, effect‐site concentration. (D) Amplitude of the first twitch of the train‐of‐four response measured by electromyography‐based neuromuscular monitoring at the adductor pollicis. Values are expressed as percentages of the reference calibrated after anesthetic induction. The reference does not represent an awake predrug baseline. The first‐twitch amplitude decreased during anesthesia and increased during emergence.

Anesthesia was maintained with remimazolam 0.5–1 mg/kg/h, remifentanil 0.25–0.30 μg/kg/min, and intermittent fentanyl (total dose, 300 μg). Pneumoperitoneum was maintained at an insufflation pressure of 10 mm Hg. No patient movement occurred, adequate surgical exposure was maintained, and the surgeon did not request neuromuscular blockade. Mechanical ventilation was provided using volume‐controlled synchronized intermittent mandatory ventilation with a tidal volume of 400 mL, a respiratory rate of 12 breaths/min, a positive end‐expiratory pressure of 5 cm H_2_O, and an inspired oxygen fraction of 0.40. Arterial carbon dioxide and oxygen tensions ranged from 33.3 to 42.1 mm Hg and from 152 to 179 mm Hg, respectively. Hemodynamics remained stable throughout anesthesia (Figure 1A). Although bispectral index values ranged from 60 to 76, the electroencephalographic spectrogram showed continuous activity dominated by delta and alpha oscillations (Figure 1B).

Surgery was completed uneventfully in 3 h 22 min, and the total duration of anesthesia was 5 h 33 min. A transversus abdominis plane block and rectus sheath block were then performed using 60 mL of 0.15% levobupivacaine (90 mg) for postoperative analgesia. After remimazolam and remifentanil were discontinued, the patient responded to verbal commands and demonstrated spontaneous swallowing and coughing. Spontaneous ventilation was confirmed with a tidal volume of 300 mL, a respiratory rate of 10 breaths/min, a minute ventilation of 3 L/min, and an end‐tidal carbon dioxide concentration of 40 mm Hg. Flumazenil 0.2 mg was then administered, followed by tracheal extubation.

Estimated effect‐site concentrations of remimazolam, remifentanil, and fentanyl, calculated using published pharmacokinetic/pharmacodynamic models [6–8], are shown in Figure 1C, and the amplitude of the first twitch of the train‐of‐four response is shown in Figure 1D. Relative to the post‐induction reference, the first‐twitch amplitude decreased to 88% during anesthesia and increased to 116% during emergence; the train‐of‐four ratio ranged from 0.96 to 1.12.

The patient was admitted to the intensive care unit for postoperative observation. Her postoperative serum creatine kinase concentration was 143 U/L. A fentanyl infusion, initiated in the operating room at 0.53 μg/kg/h, was increased to 0.64 μg/kg/h after respiratory stability was confirmed and was continued until postoperative day 1. Pain scores remained between 1 and 3 on a numerical rating scale. Supplemental oxygen was gradually reduced and discontinued on postoperative day 1 when oxygen saturation measured by pulse oximetry was 96% while breathing room air. No resedation or respiratory compromise was observed. A structured postoperative interview for intraoperative awareness was not performed; however, the patient did not spontaneously report intraoperative awareness or recall during the postoperative hospital stay. She left the intensive care unit on postoperative day 1 and was discharged home on postoperative day 5 without complications.

## 3. Discussion

This case illustrates the feasibility of a planned strategy to avoid neuromuscular blocking agents during laparoscopic surgery in a selected patient with IMNM and marked preexisting muscle weakness. Her marked baseline weakness and dysarthria raised concern that even a small degree of residual neuromuscular blockade could be clinically consequential. Low‐dose rocuronium with quantitative neuromuscular monitoring and planned sugammadex reversal was considered an alternative. The agreed initial plan was to attempt intubation and surgery without a neuromuscular blocking agent, with low‐dose rocuronium immediately available if intubation, patient immobility, or surgical exposure proved inadequate. In this patient, smooth tracheal intubation, immobility during pneumoperitoneum, adequate surgical exposure, and uncomplicated postoperative respiratory recovery were achieved without neuromuscular blockade.

Remimazolam was selected as the hypnotic for its short duration of action and reversibility with flumazenil [5], features relevant to the planned emergence and extubation strategy in a patient with limited neuromuscular reserve. Given the reported pharmacodynamic interaction between remimazolam and remifentanil [9], the continuous remifentanil infusion may have contributed to the absence of coughing or movement during tracheal intubation and to the maintenance of intraoperative immobility. Although bispectral index values ranged from 60 to 76, the electroencephalographic spectrogram showed continuous activity dominated by delta and alpha oscillations, consistent with previously reported patterns during remimazolam anesthesia [10]. No patient movement or hemodynamic response suggesting inadequate anesthesia was observed. Although a structured postoperative interview was not performed, the patient did not spontaneously report intraoperative awareness or recall.

Flumazenil was administered only after responsiveness to verbal commands, airway reflexes, and adequate spontaneous ventilation had been confirmed. Its purpose was to minimize residual hypnotic effects of remimazolam before extubation; it was not used to treat delayed awakening or inadequate ventilation. No resedation or respiratory compromise occurred postoperatively despite continued fentanyl analgesia. Although resedation after flumazenil remains possible [11], antagonism of residual hypnotic effects may be useful in patients with limited neuromuscular reserve.

Relative to the postinduction reference, the first‐twitch amplitude decreased to 88% during anesthesia and increased to 116% during emergence; the train‐of‐four ratio ranged from 0.96 to 1.12. A decrease in the first‐twitch response without a change in the train‐of‐four ratio has been reported during remimazolam anesthesia [12]. Calibration was performed after remimazolam and remifentanil had been initiated; thus, the reference was a postinduction value and not an awake predrug baseline. In addition, the first‐twitch amplitude at the adductor pollicis does not necessarily reflect generalized skeletal‐muscle tone or abdominal‐wall relaxation. Accordingly, these measurements should be viewed only as physiologic observations and do not establish that remimazolam provided clinically sufficient surgical relaxation.

Previous reports have described anesthetic management of IMNM using neuromuscular blocking agents followed by reversal [13, 14], whereas Escher et al. reported anesthesia without neuromuscular blockade using a supraglottic airway for nonlaparoscopic surgery [15]. Remimazolam–remifentanil general anesthesia without neuromuscular blocking agents has also been reported in patients with amyotrophic lateral sclerosis undergoing percutaneous endoscopic gastrostomy with tracheal intubation [16]. The present case extends these observations to a patient with IMNM undergoing tracheal intubation and laparoscopic pneumoperitoneum without neuromuscular blockade. This approach may be particularly relevant in patients with marked baseline weakness, in whom residual neuromuscular blockade could further compromise postoperative respiratory function. However, the same marked weakness that increased concern about residual blockade may also have facilitated immobility and acceptable surgical conditions, limiting extrapolation to patients with greater muscle strength or to procedures requiring more profound relaxation. Pulmonary function testing and formal assessment of respiratory muscle strength were not performed, which limited objective evaluation of the patient’s preoperative respiratory reserve.

In conclusion, laparoscopic surgery was completed without neuromuscular blocking agents under remimazolam‐based total intravenous anesthesia in a patient with IMNM and marked preexisting muscle weakness. Smooth tracheal intubation, immobility during pneumoperitoneum, acceptable surgical exposure, and uncomplicated postoperative respiratory recovery were achieved, with low‐dose rocuronium available as rescue treatment.

## Author Contributions

Yuka Kobayashi participated in the anesthetic management of the patient, collected the clinical data, and drafted the manuscript. Ryo Wakabayashi supervised the anesthetic management of the patient, conceived the report, analyzed and interpreted the clinical and physiologic data, prepared the figure, and critically revised the manuscript. Kanji Uchida supervised the work and critically revised the manuscript.

## Funding

This work received no specific grant from any funding agency in the public, commercial, or not‐for‐profit sectors and was conducted as part of the authors’ employment at The University of Tokyo Hospital.

## Disclosure

All authors have read and approved the final version of the manuscript.

## Consent

Written informed consent was obtained from the patient for publication of this case report and the accompanying figure.

## Conflicts of Interest

Ryo Wakabayashi reports receiving Grants‐in‐Aid for Scientific Research (KAKENHI) from the Japan Society for the Promotion of Science (24K23046 and 25K19832) and support from the University of Tokyo GAP Fund Program, unrelated to the present work. Ryo Wakabayashi is an inventor on a pending institution‐owned patent related to electroencephalographic time–frequency analytic approaches for detecting cerebral hypoperfusion and has received no inventor compensation. Kanji Uchida reports receiving Grants‐in‐Aid for Scientific Research (KAKENHI) from the Japan Society for the Promotion of Science (23K24425), research funding from Nihon Kohden Corporation and Nipro Corporation through separate collaborative research agreements, and speaker honoraria from AIR WATER INC., Baxter Limited, B. Braun Aesculap Japan Co., Ltd., Covidien Japan Co., Ltd., Daiichi Sankyo Co., Ltd., ICU Medical, Inc., Maruishi Pharmaceutical Co., Ltd., and Otsuka Pharmaceutical Co., Ltd., all unrelated to the present work. The other author declares no conflicts of interest.

## Supporting Information

Additional supporting information can be found online in the Supporting Information section.

## Supporting information


**Supporting Information** CARE checklist.

## Data Availability

The data supporting the findings of this case report are available from the corresponding author upon reasonable request. The data are not publicly available because of applicable ethical and privacy restrictions.
